# The use of porcine acellular dermal matrices in implant-based breast reconstruction: A systematic literature review

**DOI:** 10.1016/j.jpra.2026.08.012

**Published:** 2026-08-19

**Authors:** Deeba Ahmed, Vivek Mukhatyar, Jason Hammer, Teresa Zhou, Rebecca Wilson

**Affiliations:** a Allergan Aesthetics, an AbbVie company, 1 Millennium Way, Branchburg, NJ, 08876, USA; b North Tees and Hartlepool NHS Trust, Hardwick Road, Stockton-on-Tees, TS19 8PE, UK

**Keywords:** Mammaplasty, Follow-up studies, Necrosis, Acellular dermis

## Abstract

**Objective:**

This systematic literature review was conducted to assess outcomes reported with the use of porcine acellular dermal matrices (ADMs), Strattice and Artia, for implant-based breast reconstruction (IBBR).

**Methods:**

Articles were identified using multi-string searches of PubMed, Embase, and Medline and targeted searches of recent reviews. Hits were screened against prespecified criteria to identify those reporting adverse events or complications with Strattice or Artia in IBBR. Proportions of breasts affected were determined for implant loss, major and minor complications, and necrosis based on cohorts reporting those outcomes and analyzed descriptively.

**Results:**

A total of 14 studies were identified; of those, three studies reported multiple cohorts, for a total of 18 observational cohorts (Strattice only, 13 cohorts; Artia only, two cohorts; both products, three cohorts). Follow-up for all cohorts ranged from 3.0 months to 51.6 months. The overall pooled proportion of breasts with implant loss was 7.6% (range, 0% to 27.5%; n = 1572 breasts, 15 cohorts). Among seven cohorts (n = 704 breasts) reporting major and minor complications, pooled rates were 9.8% (range, 0% to 28.6%) and 10.8% (range, 7.5% to 27.5%) for major and minor complications, respectively. The pooled rate of skin necrosis was 4.0% (range, 0.9% to 20.4%; n = 796 breasts, seven cohorts). Pooled implant loss and necrosis rates for Strattice procedures were lower than the overall pooled rates.

**Conclusions:**

In this systematic literature review, pooled rates of implant loss, major complications, minor complications, and skin necrosis for IBBR using the porcine ADMs Strattice and Artia were comparable with previously reported rates for ADM-assisted IBBR. Additional studies are needed to assess outcomes with Artia in IBBR.

## Introduction

Implant-based breast reconstruction (IBBR) is the most frequently performed procedure for breast reconstruction in the US and worldwide.^1^^,^^2^ In post-mastectomy breast reconstruction, implants historically were placed subcutaneously.^3^^,^^4^ However, subcutaneous (or prepectoral) placement was associated with high complication rates, in particular, risk of capsular contracture and implant extrusion.^4^^,^^5^ The total submuscular technique was subsequently developed, placing the implant beneath pectoralis major and/or serratus anterior.^6^ This technique, despite being used for many years, is also subject to complications, including poor lower pole coverage and animation deformity.^7^, ^8^, ^9^

The adoption of acellular dermal matrices (ADMs) for use in breast reconstruction represented a pivotal advance for IBBR.^2^^,^^3^^,^^10^^,^^11^ ADMs provide several advantages, including the ability to create a larger subpectoral pocket, improved ability to define implant placement relative to the inframammary fold, more rapid tissue expansion and complete integration, and added protection between the implant and the mastectomy skin flap.^2^^,^^12^^,^^13^ Use of ADMs in IBBR can also improve aesthetic results and reduce rates of capsular contracture, which typically requires revision surgery.^10^^,^^12^^,^^14^^,^^15^ Along with the improvement in mastectomy technique and implant manufacturing, ADMs have facilitated a renewed development of prepectoral approaches to IBBR.^16^^,^^17^ By 2022, approximately half of all breast reconstructions conducted in the US employed ADMs^1^; a similar rate has been reported for the UK.^13^ There are reports ADM use may be associated with increased risk of postoperative infection, skin necrosis, and seromas.^18^ However, outcomes are not consistent across studies. In a large cohort study, rates of seroma were significantly lower for IBBR with ADM versus submuscular implant without ADM,^19^ and in meta-analyses comparing IBBR with versus without ADM, similar overall risks of infections, seroma, total complications, and implant loss have been reported.^18^^,^^20^ In those analyses, variability between included trials was substantial.^18^^,^^20^ Additionally, there may be a differential risk of complications among different ADM products.^2^^,^^3^^,^^21^, ^22^, ^23^

ADMs are derived from mammalian (human, bovine, or porcine) dermal tissue that is processed to remove cells and other potentially antigenic material, minimizing the risk of inflammatory and/or immunogenic responses. The resulting collagen-based infrastructure forms a flexible scaffold that is thought to encourage ingress of host cells, revascularization, and tissue regeneration.^2^^,^^11^ Human-derived ADMs are not available in Europe, due in part to extensive regulatory requirements.^24^ For this reason, European breast reconstructions have employed porcine and bovine ADMs, while human ADMs are more frequently used in the US.^2^

Strattice and Artia (Allergan Aesthetics, an AbbVie company, Irvine, CA, USA) are porcine-derived ADMs, introduced in 2008 and in 2015, respectively.^25^^,^^26^ They are cleared by the US Food and Drug Administration for use to reinforce soft tissue where weakness exists and for surgical repair of damaged or ruptured soft tissue membranes, including those requiring the use of reinforcing or bridging material to achieve desired surgical outcomes.^27^^,^^28^ Both products are designed to provide a scaffold and to facilitate incorporation into recipient tissue and associated cellular and microvascular ingrowth^27^^,^^28^ and have been processed to remove terminal disaccharide (galactose-α−1,3-galactose) residues that are believed to contribute to immunogenic responses.^25^ In addition, Artia is processed to provide 3-mm die-punched holes (fenestrations) that are thought to encourage ingrowth/colonization by surrounding cells and tissues.^11^^,^^26^^,^^29^ Artia is sterilized using electron-beam irradiation to minimize potentially antigenic changes in matrix proteins. To date, no review of outcomes for immediate IBBR procedures using these porcine-derived ADMs has been published.

The primary objective of this systematic literature review was to summarize information in the published literature regarding adverse events or complications encountered after breast reconstruction with two porcine ADMs, Strattice and Artia. Outcomes of interest in the analysis were implant loss, major (ie, requiring readmission/reoperation) and minor (managed without surgical intervention) complication rates, and skin necrosis. A secondary objective was to summarize patient-reported outcomes using standardized assessment following IBBR using either Strattice or Artia ADMs; however, few publications reporting standardized assessment outcomes were identified; therefore, the secondary objective could not be addressed.

## Methods

This systematic literature review was conducted in accordance with the 2020 Preferred Reporting Items for Systematic Literature Reviews and Meta-Analysis (PRISMA) guidelines.^30^ A Population, Intervention, Comparators, Outcomes, and Study (PICOS) design framework (Table 1) was implemented, describing eligibility criteria for studies to be targeted for inclusion.

**Table 1:** Population, Intervention, Comparators, Outcomes, and Study (PICOS) design criteria for porcine ADM systematic literature analysis.

Characteristic	Criteria
Population	Women undergoing breast reconstruction Any indication for breast reconstruction Age ≥18 years
Interventions	Strattice reconstructive tissue matrix Artia reconstructive tissue matrix
Outcomes	Adverse events (major) Adverse events (minor) Capsular contracture Patient-reported outcomes (using standardized assessments)
Comparator	Any or none
Study design	Any study design
Date limit	None
Other	Any language Exclusions: Studies conducted in animals Studies reporting data from case reports, narrative review articles, editorials, comments, and correspondence Other systematic reviews and meta-analyses (to be used only to identify original articles)

ADM, acellular dermal matrix.

### Search strategy

Comprehensive multi-string search strategies were employed to interrogate key databases (PubMed, Medline, and Embase). Searches were constructed through the ProQuest platform, which automatically removes duplicate records during searches, using an adapted version of filters published by the Scottish Intercollegiate Guidelines Network. As with the PICOS criteria, broad initial search criteria were used to facilitate capture of all relevant published sources. Initial search results were then interrogated using a targeted search to identify articles describing the use of prepectoral implantation or subpectoral implantation employing Strattice or Artia. Details of each search string, along with the number of hits returned, are summarized in Table 2. A separate search targeted potentially relevant recent systematic reviews and network meta-analyses. Reference lists from the identified review articles were hand-searched for additional relevant primary articles not identified via the database searches.

**Table 2:** Database search strategy and search strings.

Search Number	Search String	Hits
S1	TI,AB(breast* AND reconstruct* OR operat*) OR TI,AB(mammaplasty OR mammoplast* OR mastoplast*)	3,437,962
S2	TI,AB(implant*)	1,166,123
S3	S1 OR S2	4,464,088
S4	TI,AB(Artia OR Strattice)	345

AB (abstract) and TI (title) denote the database fields to be searched.

### Study selection

Abstracts and bibliographic information for all references identified in the searches were uploaded to the EndNote bibliographic platform for de-duplication and count correction. Full citations for eligible references/abstracts were then transferred to a systematic literature review platform (based on Microsoft Excel) for title and abstract screening. Titles and abstracts were independently assessed by two reviewers against the prespecified PICOS criteria; those not meeting eligibility criteria were excluded. Full texts of references considered potentially eligible were reviewed to determine eligibility, and any discrepancies between reviewers were resolved by a third senior reviewer. Rationale was documented for each article excluded.

### Data extraction and collection

The prespecified list of data targeted for extraction included publication and basic study information, patient characteristics, and outcomes. The list was modifiable as the literature review progressed. Initial extraction of available and relevant data from each study was conducted by a single reviewer. Completeness and accuracy of the extracted data were then assessed by a second reviewer. All extracted data were collated into Excel tables for subsequent analysis. Two of the included studies reported multiple cohorts^31^^,^^32^; cohorts were included separately and data were assessed at the cohort level in this analysis. For studies including evaluations of ADMs other than Strattice or Artia, only data pertaining to Strattice or Artia were recorded.

### Data analysis

All retained studies were assessed for study quality using the Newcastle-Ottawa Scale (NOS) for case-control and cohort studies.^33^, ^34^, ^35^ Study assessment was conducted by one reviewer and checked/verified by a second reviewer, and any discrepancies were adjudicated by consensus. The scoring range for the NOS is 0–9; studies scoring 0–4 were considered low quality, and those scoring 5–9 were considered high quality.

All results derived from the extracted data were summarized descriptively. No formal inferential analyses were conducted. Pooled proportions of breasts with implant loss, major complications, minor complications, and skin necrosis were calculated overall (including all cohorts reporting that outcome, weighted by number of breasts at risk for each outcome), and for Strattice and Artia cohorts separately where sufficient data were available. For each outcome, pooled proportions of breasts affected were calculated as: the sum of each included cohort of number of breasts affected, divided by the total number of breasts at risk in the analysis. Where data were available from two or fewer studies, proportions of breasts affected were reported by study with no pooling. Because the number of patients was not reported for 28% of cohorts, outcomes were not calculated as proportions of patients. One cohort included in the implant loss analysis did not have sample size reported in number of breasts at risk; in that case, number of breasts at risk was imputed based on the number of patients. Proportions of breasts affected reported for cohorts were taken from the source articles and in no case were calculated based on an imputed number.

## Results

### Study selection and data extraction

**Fig. 1:**
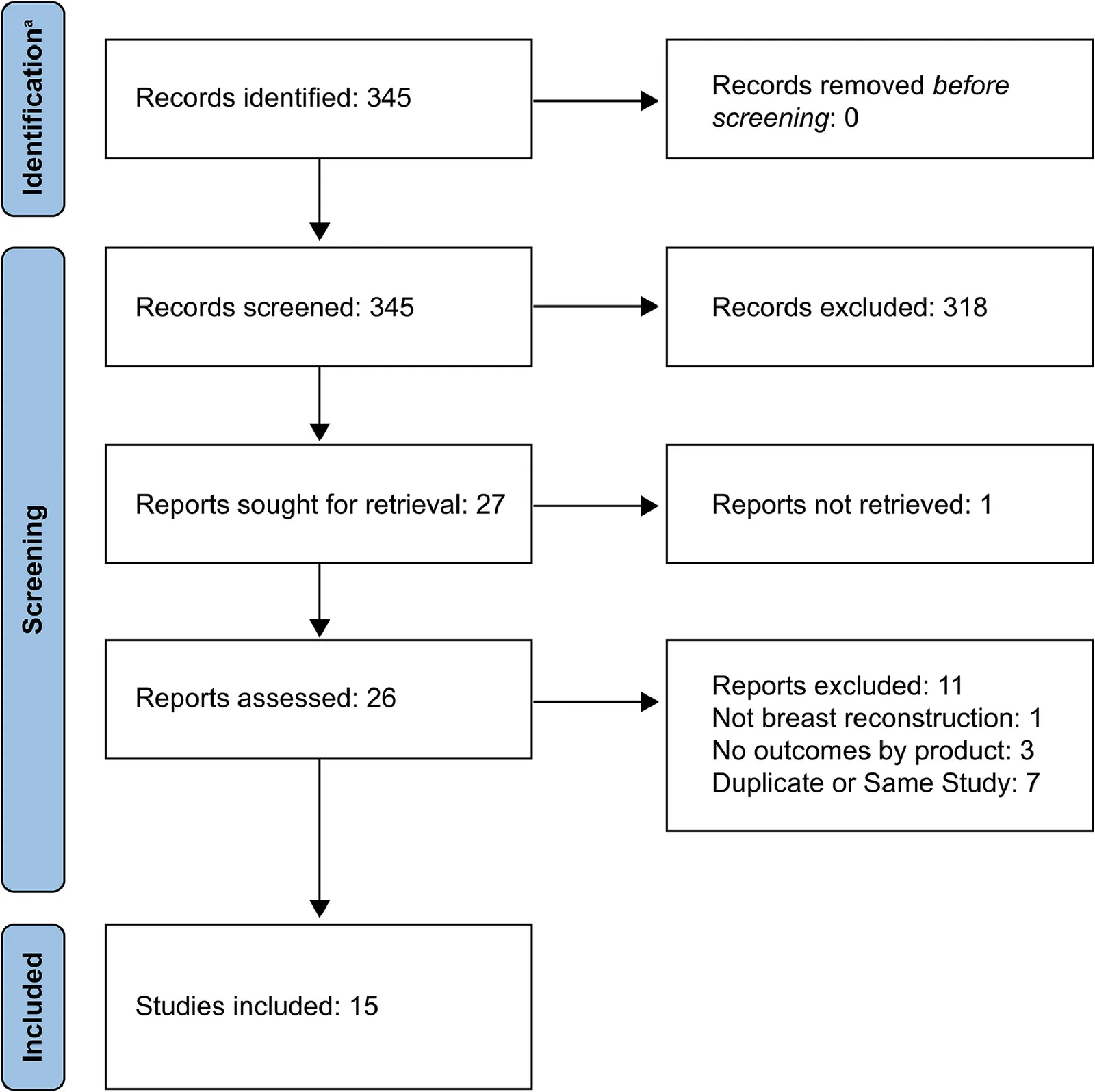
PRISMA flow diagram for retained records. ^a^Database searches were conducted using the ProQuest platform, which automatically deletes duplicate records; no records were identified via non-database sources.

All interrogations of PubMed, Medline, and Embase were conducted on November 9, 2023. The initial search returned a total of 345 unduplicated records. Following exclusions during title/abstract review (n = 318) and full-text review (n = 12, plus 1 article not retrieved), 14 articles were retained for data extraction (Fig. 1).

### Characterization of retained studies

The 14 studies retained for analysis are summarized in Table 3. Study periods ranged from 2010 to 2021, and corresponding articles were published from 2012 through 2023. The leading country of origin was the United Kingdom (n = 8), followed by the United States (n = 2), Germany (n = 2), Ireland (n = 1), and Austria (n = 1). The NOS study quality assessment for each retained study ranged from 2 to 7 (**Supplemental Table S1**). Most studies (n = 10) scored from 5 to 7, at the middle/lower part of the “high quality” range (5–9).

**Table 3:** Summary of retained studies.

Author, Year	Title	Time Horizon	Country
Baker, 2018^36^	A prospective comparison of short-term outcomes of subpectoral and prepectoral Strattice-based immediate breast reconstruction	Mar 2016 to Apr 2016	United Kingdom
Ball, 2017^37^	A direct comparison of porcine (Strattice) and bovine (SurgiMend) acellular dermal matrices in implant-based immediate breast reconstruction	Oct 2011 to Mar 2016	United Kingdom
Dave, 2021^31^	Risk factors for complications and implant loss after prepectoral implant-based immediate breast reconstruction: medium-term outcomes in a prospective cohort	Jun 2013 to Mar 2019	United Kingdom
Ferede, 2013^38^	Porcine acellular dermal matrix (Strattice)-based immediate breast reconstruction: the South Eastern breast cancer experience	2010 to 2012	Ireland
Fung, 2014^39^	The matrix: Strattice vs XCM in immediate implant-based breast reconstruction	NR	United Kingdom
Hashmi, 2017^40^	Reconstructive breast surgery: compatibility of Strattice matrix with radiation therapy	2010 to 2015	United Kingdom
Highton, 2017^32^	Prepectoral implant-based breast reconstruction	Jun 2013 to Feb 2017	United Kingdom
Kaushik, 2012^41^	Initial experience with the use of porcine acellular dermal matrix for breast reconstruction	NR	United Kingdom (Implied)
Kiechle, 2012^42^	Use of acellular dermis (Strattice) in problematic cases of breast reconstructive surgery	Mar 2011 to NR	Germany (Implied)
Mitchell, 2013^43^	Porcine acellular dermis–assisted breast reconstruction: influence of adjuvant radiotherapy on complications and outcomes	Jan 2009 to May 2013	United States
Reitsamer, 2012^44^	Technical results and complication rates after skin-sparing and nipple-sparing mastectomy and immediate implant-based reconstruction using a porcine tissue matrix Strattice for implant coverage	NR	Austria
Schüler, 2021^45^	Postoperative complications in breast reconstruction with porcine acellular dermis and polypropylene meshes in subpectoral implant placement	Nov 2010 to Jun 2015	Germany
King, 2023^46^	An evaluation of the relative safety of Artia porcine acellular dermal matrix in the setting of implant-based breast reconstruction	Jan 2015 to Mar 2021	United States
Wilson, 2023^19^	Breast reconstruction outcomes with and without Strattice: long-term outcomes of a multicenter study comparing Strattice immediate implant breast reconstruction with submuscular implant reconstruction	Jan 2009 to Dec 2015	United Kingdom

NR, not reported.

A total of 18 cohorts within the 14 studies were identified for descriptive analysis (Table 4). Eleven of the 14 cohorts reported number of breasts at risk (n = 1582 breasts); 13 cohorts reported number of patients (n = 875 individuals). A total of 13 cohorts included patients treated with Strattice exclusively (n = 1211 breasts), two with Artia exclusively (n = 139 breasts), and three with both products (n = 232 breasts). Classified by surgical approach, six cohorts each received prepectoral (n = 609 breasts) or subpectoral (n = 247 breasts) implants only, two received both prepectoral and subpectoral implants, and approach was unreported in four cohorts. Direct/Immediate reconstruction was employed in nearly all cases where data were reported. For the 14 cohorts that reported follow-up time, durations ranged from 3.0 months to 51.6 months.

**Table 4:** Characterization of included cohorts.

Author, Year	Follow-up (Months)	Product (Approach)	Age (Years)	Sample Size (People)	Sample Size (Breasts)	Surgical Indication (% Patients, Direct/ Immediate Reconstruction)	Surgical Indication (% Patients, Delayed Reconstruction)	Surgical Indication (% Patients, Revised Reconstruction)
Baker, 2018^36^	3.0	Strattice (PrePec)	47.5	28	43	100%	NR	0%
	3.0	Strattice (SubPec)	48	12	19	100%	NR	0%
Ball, 2017^37^	23.4	Strattice (SubPec)	44	19	30	68%	NR	NR
Dave, 2021^31^	21.0	Strattice (PrePec)	NR	NR	293	100%	NR	NR
	21.0	Strattice and Artia (PrePec)	NR	NR	66	100%	NR	NR
	21.0	Artia (PrePec)	NR	NR	41	100%	NR	NR
Ferede, 2013^38^	NR	Strattice (NR)	NR	NR	35	100%	0%	0%
Fung, 2014^39^	NR	Strattice (NR)	NR	NR	13	100%	0%	0%
Hashmi, 2017^40^	27.0	Strattice (SubPec)	52	76	NR	50%	47%	NR
Highton, 2017^32^	16.2	Strattice or Artia (PrePec)^a^	44	71	113	100%	NR	NR
	16.2	Strattice or Artia (PrePec)^b^	44	35	53	NR	NR	100%
Kaushik, 2012^41^	4.0	Strattice (NR)	NR	10	NR	90%	NR	10%
Kiechle, 2012^42^	NR	Strattice (SubPec)	NR	8	NR	NR	NR	100%
Mitchell, 2013^43^	36.2	Strattice (SubPec)	51	103	158	100%	NR	NR
Reitsamer, 2012^44^	NR	Strattice (NR)	NR	22	27	100%	NR	0%
Schüler, 2021^45^	17.8	Strattice (SubPec)	52.3	34	40	NR	NR	NR
King, 2023^46^	17.2	Artia (Both)	49.2	63	98	NR	NR	0%
Wilson, 2023^19^	51.6	Strattice (Both)	52	394	553	100%	NR	NR

NR, not reported; PrePec, prepectoral implant placement; SubPec, subpectoral implant placement.

a Direct reconstruction group.

b Revised reconstruction group.

### Summary of outcomes

A total of 15 cohorts (n = 1572 breasts) contributed data on implant loss, including 10 Strattice cohorts (n = 1201 breasts) and two Artia cohorts (n = 139 breasts). The overall pooled mean proportion of breasts that experienced implant loss across cohorts with available data was 7.6% (range, 0% to 27.5%; Table 5). Rates of implant loss were 14% or less for all but two cohorts; the two cohorts with greater rates of implant loss included one Artia cohort with a mean follow-up of 17.2 months (n = 98 breasts; 21.0% implant loss),^46^ and one Strattice cohort with a 17.8-month average follow-up (n = 40 breasts; 27.5% implant loss).^45^ The pooled mean proportion of breasts with Strattice procedures that experienced implant loss was 7.6% (range, 0% to 27.5%). Implant loss was reported in 2.4% (1/41) of breasts and 21.4% (21/98) of breasts in the two Artia cohorts, respectively. Six of the 15 cohorts used a prepectoral approach only (n = 609 breasts, both Strattice and Artia), and among those cohorts, 3.3% of breasts had implant loss (range, 0% to 4.7% Three cohorts used a subpectoral approach only (n = 217 breasts; all Strattice); proportions with implant loss ranged from 0% to 27.5%. No clear relationship between follow-up duration and reported rate of loss was apparent (Table 5).

**Table 5:** Outcome summary: implant loss.

Author, Year	Follow-up (Months)	Product (Approach)	Number of Patients at Risk	Number of Breasts at Risk	Implant Loss (% Breasts)	Implant Loss (n Breasts)
Baker, 2018^36^	3.0	Strattice (PrePec)	28	43	4.7%	2
	3.0	Strattice (SubPec)	12	19	0%	0
Dave, 2021^31^	21.0	Strattice (PrePec)	NR	293	3.4%	10
Ferede, 2013^38^	NR	Strattice (NR)	NR	35	14%	5
Fung, 2014^39^	NR	Strattice (NR)	NR	13	8%	1
Kaushik, 2012^41^	4.0	Strattice (NR)	10	NR	0%	0
Mitchell, 2013^43^	36.2	Strattice (SubPec)	103	158	8.2%	13
Reitsamer, 2012^44^	NR	Strattice (NR)	22	27	7.4%	2
Schüler, 2021^45^	17.8	Strattice (SubPec)	34	40	27.5%	11
Dave, 2021^31^	21.0	Artia (PrePec)	NR	41	2%	1
King, 2023^46^	17.2	Artia (Both)	63	98	21%	21
Dave, 2021^31^	21.0	Strattice and Artia (PrePec)	NR	66	3%	2
Highton, 2017^32^	16.2	Strattice or Artia (PrePec)^a^	71	113	4.4%	5
Highton, 2017^32^	16.2	Strattice or Artia (PrePec)^b^	35	53	0%	0
Wilson, 2023^19^	51.6	Strattice (Both)	394	553	8.5%	47

NR, not reported; PrePec, prepectoral implant placement; SubPec, subpectoral implant placement.

a Direct reconstruction group.

b Revised reconstruction group.

Seven cohorts (n = 704 breasts) contributed data on major and minor complications; two included Strattice procedures only (n = 333 breasts), two included Artia only (n = 139 breasts), and three included both Strattice and Artia procedures (n = 232 breasts; Table 6). In all studies, major complications were defined as those requiring readmission/reoperation, and minor complications were those managed without surgical intervention.^31^^,^^32^^,^^45^^,^^46^ Overall, the proportion of breasts with major complications was 9.8% (range, 0% to 28.6%), and the proportion with minor complications was 10.8% (range, 7.5% to 27.5%). The two Strattice cohorts had major complication rates of 6.1% (18/293) and 27.5% (11/40) of breasts, respectively, and minor complication rates of 11.3% of (33/293) and 27.5% (11/40) of breasts, respectively. In the two Artia cohorts, major complications were reported in 9.8% (4/41) and 28.6% (28/98) of breasts at risk, and minor complications were reported in 9.8% (4/41) and 9.2% (9/98). Five of the seven cohorts used a prepectoral approach (n = 566 breasts), and among those cohorts, 5.3% of breasts had major complications (range, 0% to 9.8%) and 9.9% had minor complications (range, 7.5% to 11.3%). One cohort reporting major or minor complications rates used a subpectoral approach (n = 40 breasts, Strattice). In that cohort, 27.5% of breasts had major complications and 27.5% had minor complications.

**Table 6:** Outcome summary: major and minor complications.

Author, Year	Follow-up (Months)	Product (Approach)	Number of Patients at Risk	Number of Breasts at Risk	Complication, Minor (% Breasts)	Complication, Minor (n Breasts)	Complication, Major (% Breasts)	Complication, Major (n Breasts)
Dave, 2021^31^	21.0	Strattice (PrePec)	NR	293	11.3%	33	6.1%	18
Schüler, 2021^45^	17.8	Strattice (SubPec)	34	40	27.5%	11	27.5%	11
Dave, 2021^31^	21.0	Artia (PrePec)	NR	41	10%	4	10%	4
King, 2023^46^	17.2	Artia (Both)	63	98	9%	9	29%	28
Dave, 2021^31^	21.0	Strattice and Artia (PrePec)	NR	66	8%	5	5%	3
Highton, 2017^32^	16.2	Strattice or Artia (PrePec)^a^	71	113	8.8%	10	4.4%	5
Highton, 2017^32^	16.2	Strattice or Artia (PrePec)^b^	35	53	7.5%	4	0%	0

NR, not reported; PrePec, prepectoral implant placement; SubPec, subpectoral implant placement.

a Direct reconstruction group.

b Revised reconstruction group.

Specific complications were reported for four cohorts: complications after Strattice procedures (one cohort; n = 40 breasts) included seroma, hematoma, wound healing disorder, suture dehiscence, wound infection, skin necrosis, abscess, revision surgery, and implant loss/reconstructive failure.^45^ Complications after Artia procedures (one cohort; n = 98 breasts) were seroma, hematoma, infection, tissue necrosis, capsular contracture, and shape deformity.^46^ In two mixed Strattice and Artia cohorts (n = 166 breasts total), complications included red breast, seroma, minor slough/infection/healing delay, skin necrosis, skin and nipple necrosis, and recalcitrant seroma leading to wound breakdown.^32^

Incidence of skin necrosis was reported in seven cohorts (n = 796 breasts at risk), including six Strattice cohorts (n = 698 breasts at risk) and one Artia cohort (n = 98 breasts at risk). Overall, the pooled mean proportion of breasts with skin necrosis was 4.0% (range, 0.9% to 20.4%; Table 7). Skin necrosis was reported in a pooled mean of 1.7% of breasts (range, 0.9% to 7.7%) in the Strattice cohorts, and was observed in 20.4% (20/98) of breasts in the Artia cohort. A single cohort providing necrosis data used a prepectoral approach (n = 43 breasts; Strattice), and in that cohort, incidence of necrosis was 4.7% of breasts. Three cohorts used a subpectoral approach only (n = 89 breasts; all Strattice); proportions with skin necrosis ranged from 3.3% to 5.3% in those cohorts.

**Table 7:** Outcome summary: skin necrosis.

Author, Year	Follow-up (Months)	Product (Approach)	Number of Patients at Risk	Number of Breasts at Risk	Skin Necrosis (% Breasts)	Skin Necrosis (n Breasts)
Baker, 2018^36^	3.0	Strattice (PrePec)	28	43	4.7%	2
	3.0	Strattice (SubPec)	12	19	5.3%	1
Ball, 2017^37^	23.4	Strattice (SubPec)	19	30	3.3%	1
Fung, 2014^39^	NR	Strattice (NR)	NR	13	8%	1
Schüler, 2021^45^	17.8	Strattice (SubPec)	34	40	5%	2
Wilson, 2023^19^	51.6	Strattice (Both)	394	553	0.9%	5
King, 2023^46^	17.2	Artia (Both)	63	98	20%	20

NR, not reported; PrePec, prepectoral implant placement; SubPec, subpectoral implant placement.

## Discussion

In this systematic review of outcomes for immediate IBBR using porcine ADMs, Strattice and Artia had rates of implant loss and skin necrosis comparable to rates previously published in ADM-assisted IBBR.^18^^,^^20^^,^^47^, ^48^, ^49^, ^50^, ^51^, ^52^, ^53^ Overall complication rates in those studies range from 12% to 23%; major and minor complication rates were not analyzed separately.^18^^,^^48^^,^^52^^,^^54^ Notably, in the current review, pooled rates of implant loss and major complications were lower in cohorts using the prepectoral technique for IBBR than the overall pooled rates. This is in line with results from recent meta-analyses reporting similar or lower rates of complications for prepectoral versus subpectoral procedures, among which 87% to 100% of included studies used ADM.^55^, ^56^, ^57^

In the current review, pooled implant loss and skin necrosis rates for Strattice procedures were lower than the overall rates; rates of major and minor complications were reported in just two Strattice cohorts. Only two studies reporting these outcomes for Artia met inclusion criteria, so no pooled proportions were calculated for that ADM. Additionally, implant loss and major complication rates for the two Artia cohorts were disparate, and indeed, variability across all cohorts was high. Two of the cohorts in the analysis (Schüler et al., 2021 [Strattice] and King et al., 2023 [Artia]) had substantially higher rates of implant loss (27.5%, 20.0%, respectively), complications (major: 27.5% and 28.6%, respectively; minor: 27.5%; Schüler et al.), and skin necrosis (20.0%; King et al.) than those reported in other cohorts.^45^^,^^46^ In discussing the high rates of complications in their study, Schüler and colleagues noted that a substantial number of patients in the Strattice cohort (23.5%) had preoperative radiation therapy, which was a significant risk factor for complications and implant loss in their study.^45^ Smoking also has been associated with complications (including implant loss and skin necrosis) in IBBR using ADM,^58^ and 26.5% of the Strattice cohort in the Schüler study were smokers.^45^ While King et al. did not address the complication rate for Artia in their study, almost half the patients in their study population had a history of smoking.^46^ The more recent availability of Artia could also be a potential confounder. That is, elevated rates of complications could reflect a learning curve for this newer ADM, as outcomes can improve as surgeons gain experience with a product.^59^^,^^60^ However, the King et al. study included a comparator arm (human-derived ADM AlloDerm [Allergan Aesthetics, an AbbVie company]), and no statistically significant differences in rates of complications (including explantation, infection, hematoma, necrosis, seroma, and capsular contracture) for Artia versus AlloDerm procedures were observed.^46^ The incidence of any complication was numerically less with Artia.^46^

The principal limitation to the literature review was the small number of retained studies, particularly for Artia alone, and consequently, the insufficiency of the extracted data to support a statistical comparison of product-specific outcomes (Strattice vs Artia), the assessment of effects of surgical approach (prepectoral vs subpectoral), or a formal assessment of heterogeneity. Conclusions are limited by variation in follow-up duration and outcome definitions and criteria, as well as the variability in outcome rates reported; all outcomes of interest were not reported for each cohort. Body mass index, a risk factor for complications in IBBR,^61^ was not assessed, as it was reported for few cohorts. Additionally, bilateral versus unilateral reconstruction rates were not clearly reported in the source articles. For some cohorts, sample size was reported either as numbers of patients or as numbers of breasts, but not both. No patient-reported outcomes were assessed in the retained cohorts. Importantly, it should be noted that this review was funded by Allergan Aesthetics, an AbbVie company, which markets both Strattice and Artia, and four of the five authors have a current or previous affiliation with the company.

## Conclusions

This systematic literature review examined outcomes associated with IBBR using the porcine ADMs Strattice and Artia. Overall, the ADMs were associated with rates of implant loss, major and minor complications, and skin necrosis comparable to those reported in previous analyses of a wider range of ADM products. Rates of implant loss and skin necrosis appear lower in Strattice reconstruction. Additional studies, particularly with Artia, are needed to comprehensively assess both clinical and patient-reported outcomes in immediate IBBR.

## Funding disclosures

Allergan Aesthetics, an AbbVie company, funded this study and participated in the study design, research, analysis, data collection, interpretation of data, reviewing, and approval of the publication. All authors had access to relevant data and participated in the drafting, review, and approval of this publication. No honoraria or payments were made for authorship. Medical writing support was provided by Stephen Collins, MSc and Kathleen M. Dorries, PhD of Peloton Advantage, LLC (an OPEN Health company) and funded by Allergan Aesthetics, an AbbVie company.

## Ethical approval

Not required.

## Data sharing

AbbVie is committed to responsible data sharing regarding the clinical trials we sponsor. This includes access to anonymized, individual and trial-level data (analysis data sets), as well as other information (eg, protocols and clinical study reports, synopses, or statistical analysis plans), as long as the trials are not part of an ongoing or planned regulatory submission.

This clinical trial data can be requested by any qualified researchers who engage in rigorous, independent scientific research, and will be provided following review and approval of a research proposal and Statistical Analysis Plan (SAP) and execution of a Data Use Agreement (DUA). Data requests can be submitted at any time after approval in the US and Europe and after acceptance of this manuscript for publication. The data will be accessible for 12 months, with possible extensions considered. For more information on the process, or to submit a request, visit the following link: https://vivli.org/ourmember/abbvie/ then select “Home.”

## Declaration of competing interest

Deeba Ahmed: is a full-time employee of AbbVie and may hold AbbVie stock.

Vivek Mukhatyar: is a former employee of AbbVie and may hold AbbVie stock.

Jason Hammer: is a full-time employee of AbbVie and may hold AbbVie stock.

Teresa Zhou: is a full-time employee of AbbVie and may hold AbbVie stock.

Rebecca Wilson: has applied for an AbbVie research grant.

## Appendix Supplementary materials

Supplemental Material List Supplemental Table S1. Newcastle-Ottawa Scale^a^ scores for retained studies Image, application 1
