# Supplementary material for: The use of porcine acellular dermal matrices in implant-based breast reconstruction: A systematic literature review
Source: JPRAS Open. 2026 Aug 19;52:253–63. doi: 10.1016/j.jpra.2026.08.012 (PMC13571755; doi:10.1016/j.jpra.2026.08.012)
Supplement: Supplementary file 1 [file mmc1.docx]

**SUPPLEMENTAL INFORMATION**

**Supplemental Table S1.** Newcastle-Ottawa Scale^a^ scores for retained studies

| **Study ID** | **NOS Score** |
| --- | --- |
| Baker, 2018 | 6 |
| Ball, 2017 | 6 |
| Dave, 2021 | 6 |
| Ferede, 2013 | 6 |
| Fung, 2014 | 2 |
| Hashmi, 2017 | 2 |
| Highton, 2017 | 6 |
| Kaushik, 2012 | 3 |
| Kiechle, 2012 | 3 |
| Mitchell, 2013 | 5 |
| Reitsamer, 2012 | 3 |
| Schüler, 2021 | 6 |
| King, 2023 | 7 |
| Wilson, 2023 | 7 |

^a^The Newcastle-Ottawa Scale evaluates cohort study quality based on selection (ie, representativeness of exposed cohort, ascertainment of exposure); comparability (of cohorts based on study design/analysis); and outcomes (ie, assessment, adequacy/duration of follow-up). One star (or point) is awarded for inclusion of specific targets; maximum score is 9 [1].

**Reference**

1.	Coding Manual for Case-control Studies. The Ottawa Hospital Research Institute: Ontario, Canada; 2021.
